# Genomorama: genome visualization and analysis

**DOI:** 10.1186/1471-2105-8-204

**Published:** 2007-06-14

**Authors:** Jason D Gans, Murray Wolinsky

**Affiliations:** 1 Biosciences Division, Los Alamos National Laboratory, Los Alamos, NM, USA

## Abstract

**Background:**

The ability to visualize genomic features and design experimental assays that can target specific regions of a genome is essential for modern biology. To assist in these tasks, we present Genomorama, a software program for interactively displaying multiple genomes and identifying potential DNA hybridization sites for assay design.

**Results:**

Useful features of Genomorama include genome search by DNA hybridization (probe binding and PCR amplification), efficient multi-scale display and manipulation of multiple genomes, support for many genome file types and the ability to search for and retrieve data from the National Center for Biotechnology Information (NCBI) Entrez server.

**Conclusion:**

Genomorama provides an efficient computational platform for visualizing and analyzing multiple genomes.

## Background

With the rapid growth in the number of sequenced genomes has come a corresponding proliferation of computational tools for viewing, comparing and searching genome sequences and annotations. Tools can be divided into two broad categories [1], database-client and stand-alone. In general, database-client tools offer static (or semi-static) visualizations of small sets of predefined genomes, while stand-alone tools allow interactive visualizations of locally stored genomes. Stand-alone tools can serve as graphical front ends for displaying the output of locally run calculations. A high level comparison of common features for these stand-alone tools [2-19] is shown in Table 1 and reveals several trends and patterns. Almost all of the tools are implemented in an interpreted language (i.e. Java, Perl, Tcl/Tk). While this provides for cross platform portability, the responsiveness (i.e. rendering speed, file loading speed) of these applications is poor. While all of the tools can display genome annotations, additional functionalities (i.e. sequence and annotation based searching, multiple sequence alignment, annotation editing, etc.) vary widely between programs.

**Table 1:** Comparing features of freely available, stand-alone genome viewers

Program	Platforms^a^	Input formats^b^	Graphic output formats^c^	Source code available	Circular view	Linear view	Real time navigation	Multiple genomes	Annotation editing and creation	Annotation searching	Sequence searching
Apollo [2]	Java	GAME XML, GFF, GBK, EMBL, FASTA	PS								
Argo [3]	Java	GFF, GBK, GENSCAN, BLAST	Printer					(2)			
Artemis [4]	Java	EMBL, GBK, FASTA, GFF	JPG, PNG					(via ACT)			
Bluejay [5]	Java	XML	Printer, SVG								
CGView [6]	Java	PTT, XML	PNG, JPG, SVG								
DNAvis [7]	Windows, Linux	GFF, FASTA									
GATA [8]	Java	GFF	PNG					(2)			
GeneViTo [9]	Java	PTT+FFN+FNA	JPG								
GenoMap [10]	Tcl/Tk	GRS	PS								
Genome2D [11]	Windows	GBK, FASTA, GLIMMER, PARADOX	Printer, WMF, BMP								
GenomeComp [12]	Perl/Tk	EMBL, GBK, FASTA	PS					(2)			
GenomePlot [13]	Tcl/Tk/Perl	tab delimited	PS, GIF, TIFF, JPG								
GenomeViz [14]	Tcl/Tk/Perl (no Windows)	tab delimited	PS								
Genome Workbench [15]	OS X, Windows, Linux	ASN.1, XML, FASTA, GFF									
Genomorama	OS X, Windows, Linux	EMBL, GBK, ASN.1, FASTA, PTT	PS, GIF								
IGB [16]	Java	GFF, FASTA, PSL, DAS	Printer								
Mauve [17]	Java	GBK, FASTA, SEQ	PNG, JPG								
SeqVISTA [18]	Java	EMBL, FASTA	JPG								
Sockeye [19]	Java	EMBL (via server), GFF	JPG								

^a^Programs that use Java, Tcl/Tk and Perl are expected to run on any operating system. ^b^Common file formats include the GenBank flat file (GBK), EMBL flat file (EMBL), nucleic acid sequence file (FASTA), general feature format (GFF) and protein table file (PTT). A complete list of genome annotation file formats can be found on the Genomorama project webpage. ^c^The graphic output format labeled "Printer" indicates direct output to an attached printer.

Not content with the performance or feature set of existing programs, we wrote Genomorama, a stand-alone tool originally developed to assist in computational signature design for bacterial and viral pathogen detection. Genomorama allows users designing DNA-based hybridization assays, such as PCR or DNA probes, to easily identify the regions of a genome targeted by a given assay. It is distinguished from existing tools by DNA hybridization-based sequence searching, its rapid execution speed, and ability to read and export a diverse set of common file formats. Despite its origins as a viewer for viral and bacterial genomes, Genomorama can also visualize large eukaryotic genomes (e.g. human chromosomes).

## Implementation

Genomorama is a software program for interactively displaying and analyzing multiple genomes. It provides a powerful yet easy to use interface that leverages the visualization power of modern computers (via OpenGL) and the substantial bioinformatic infrastructure provided by the NCBI (via the NCBI C toolkit). Genomorama is written in portable, highly optimized C++ and comes in three "flavors" that allow it to run natively on (most) modern operating systems: OS X (using Carbon), Microsoft Windows (using the Microsoft Foundation Classes) and Linux (using Motif). The Motif version allows any X-windows client that supports OpenGL to remotely run Genomorama. Executables and source code are freely provided for all flavors.

## Results and discussion

To visualize and compare annotated genome features at all relevant size scales, genomes are displayed on the computer screen as linear, scale-dependent maps. The user interacts with a map using the mouse, keyboard and scroll bars. Semantic zooming [20] is used to display genomic features which occur at a wide range of scales, i.e. ~10^5 ^bases for a mammalian gene, ~10^4 ^bases for a pathogenicity island, ~10^3 ^bases for a bacterial gene, ~10^2 ^bases for a tRNA, ~10^1 ^bases for a transcription factor binding site and 10^0 ^for a single nucleotide polymorphism. Optional 2D graphs, including %G+C, GC skew (automatically computed from the genome sequence) and external data sets (provided by the user in a separate file), can be superimposed on genome maps. Publication quality, WYSIWYG ("What You See Is What You Get") images can be saved in either GIF or PostScript formats.

**Figure 1:**
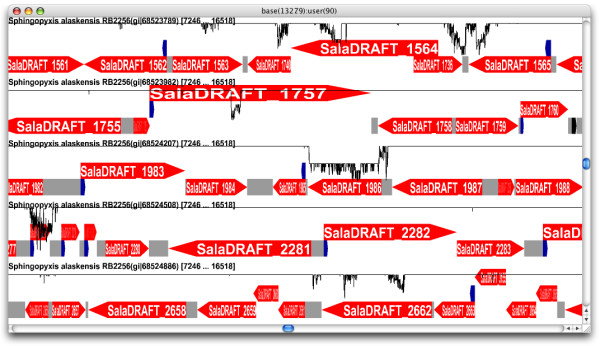
**Genomorama can load and display the multiple annotated contigs stored in a whole genome shotgun GBK file**. This screen shot shows five contigs from *Sphingopyxis alaskensis *RB2256 (extracted from the NCBI [21] file wgs.AAIP.1.gbff) and the associated sequence quality scores (from the NCBI [21] file wgs.AAIP.1.qscore). Quality scores are proportional to the negative log of the probability that a given base has been incorrectly assigned as an A, T, G or C and are shown as black plots superimposed over each contig track. The value of a quality score for each track is interactively displayed on the menu bar as a user specified score [i.e. "user(90)"] for the annotation track and base currently selected by the cursor.

Genome annotations and sequences are available in a large number of file formats and Genomorama can read a substantial subset of these formats, including GenBank (GBK), European Molecular Biology Laboratory (EMBL), Abstract Syntax Notation One (ASN.1), Protein Table (PTT) and FASTA. Unlike existing programs, Genomorama can read the multi-part GBK, EMBL and ASN.1 files used to store annotations and sequence for partially assembled sequences for both prokaryotic and eukaryotic organisms. The ability to load multipart annotation files allows access to preliminary annotation information provided by sequencing centers during the whole genome shotgun sequencing of an organism (these files are available from the NCBI ftp site [21]). A screen shot of five contigs and associated sequencing quality scores from the genome *Sphingopyxis alaskensis *RB2256 is shown in Figure 1.

**Figure 2:**
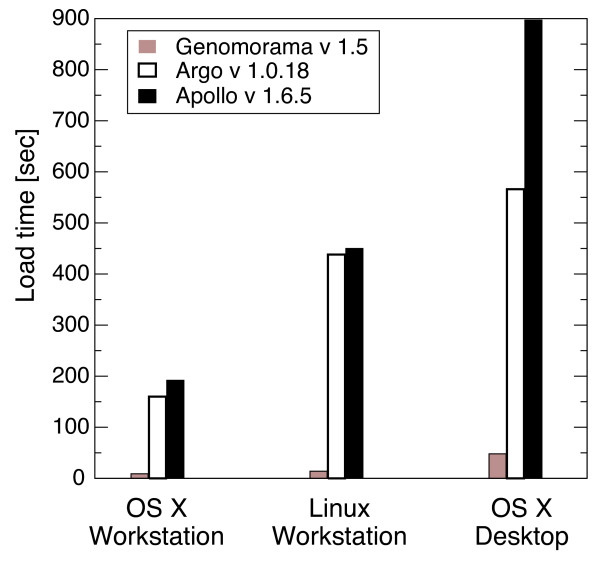
**Comparing the time to load human chromosome 1**. The time to load *Homo sapiens *chromosome 1 is used to compare the performance of Genomorama and two Java based tools: Apollo [2] and Argo [3]. The time to load the GBK file [GenBank:NC_000001.9] from the local hard drive is shown for three computing platforms: a high-end OS X 10.4.8 workstation (dual 3 Ghz Intel Xeon CPUs, 3 GB ram, Java 1.5.0), a mid-range Linux Red Hat 4.0.1 workstation (dual 2.4 GHz Intel Xeon CPUs, 1 GB ram, Java 1.4.2) and low-end OS X 10.3.9 desktop (single 1.8 GHz G5 PowerPC CPU, 512 MB ram, Java 1.4.2). The Java-based programs were run from the command line with the arguments "-Xms32m -Xmx1024m" to increase the amount of memory allowed to the Java virtual machine. Providing Java with more than 1 GB of memory did not improve performance (results not shown). Each program loaded the genome file twice (to ensure fair OS disk caching) and the second load time is reported. For all platforms, Genomorama loads the genome file more than an order of magnitude faster than either of the Java-based programs.

Genomorama can load large (> 10^8 ^bases) genomes. Support for large genomes is crucial for visualizing entire eukaryotic chromosomes. A comparison between loading times for Genomorama and two Java-based visualization tools is shown in Figure 2. Conservative memory usage and efficient C++ implementation enable Genomorama to load the sequence and annotations for human chromosome 1 substantially faster (more than an order of magnitude) than either of the Java-based programs on a range of desktop computers.

To assist in experimental design and analysis, Genomorama provides DNA hybridization-based searches to identify probe binding locations and PCR amplification products. Given a pair of PCR primers, Genomorama will display all corresponding PCR amplicons from a target sequence. Both traditional PCR primer and Padlock probe [22] queries are supported. These searches employ a sequence similarity criteria defined by DNA melting temperature [23-28], which allows for non-Watson and Crick base pairing (but currently not gaps or DNA bulges), and an optional number of exact matching bases at the 3' end of each primer. All possible combinations of the forward and reverse PCR primers are tested (i.e. forward-reverse, reverse-forward, forward-forward and reverse-reverse). In contrast, existing in-silico PCR tools are either inflexible (i.e. require a preconfigured server) [29] or rely on heuristic similarity measures (i.e. number of mismatches between primer and template) [30,31].

**Figure 3:**
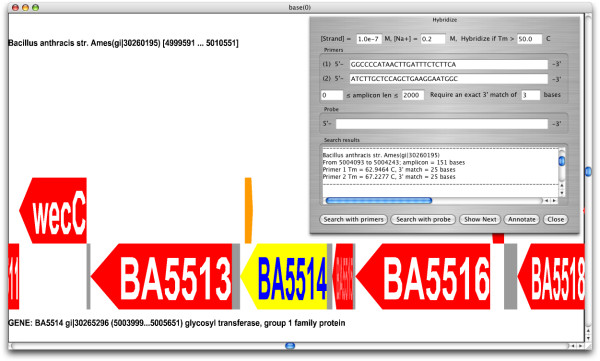
**Genomorama supports sequence searching with PCR primers**. The genomic neighborhood of the amplicon (shown in orange) produced by the *B. anthracis *[GenBank:NC_003997.3] chromosomal specific PCR primers, M.Ctg032 [32]. The amplicon is contained within a glycosyl transferase (show in yellow). The amplicon annotation was added to the genome by selecting the "annotate" button on the Hybridize dialog box.

Genomorama also performs primer prediction by computing all potential forward and reverse PCR primers that satisfy primer length, melting temperature, %G+C and heuristic base composition requirements. An example of PCR primer based searching, using the *B. anthracis *specific primers [32], is shown in Figure 3. Finally, sequence searching (both exact and hybridization based) is sensitive to the topology of the target DNA molecule (i.e. either linear or circular) and, as a result, can identify query matches that span the start/stop (i.e. nucleotide 0) of circular genomes.

## Conclusion

Genomorama is an easy to use computational tool for a number of genome comparison tasks, including real time display of multiple genomes, high quality output and novel hybridization based sequence searching.

## Availability and requirements

• **Project name: **Genomorama

• **Project homepage: **

• **Operating systems: **OS X, Windows, Linux

• **Programming language: **C++

• **License: **Freely available

• **Any restrictions on use by non-academics**: No

## Authors' contributions

JG wrote the program and documentation. MW oversaw the development process. Both authors prepared and approved the manuscript.
